# Acute and chronic effects by nicotine on striatal neurotransmission and synaptic plasticity in the female rat brain

**DOI:** 10.3389/fnmol.2022.1104648

**Published:** 2023-01-12

**Authors:** Erika Lucente, Bo Söderpalm, Mia Ericson, Louise Adermark

**Affiliations:** ^1^Integrative Neuroscience Unit, Department of Pharmacology, Institute of Neuroscience and Physiology, The Sahlgrenska Academy, University of Gothenburg, Gothenburg, Sweden; ^2^Addiction Biology Unit, Department of Psychiatry and Neurochemistry, Institute of Neuroscience and Physiology, The Sahlgrenska Academy, University of Gothenburg, Gothenburg, Sweden

**Keywords:** dopamine, electrophysiology, endocannabinoid, female, LTD, nicotine, striatum

## Abstract

**Introduction:**

Tobacco use is in part a gendered activity, yet neurobiological studies outlining the effect by nicotine on the female brain are scarce. The aim of this study was to outline acute and sub-chronic effects by nicotine on the female rat brain, with special emphasis on neurotransmission and synaptic plasticity in the dorsolateral striatum (DLS), a key brain region with respect to the formation of habits.

**Methods:**

*In vivo* microdialysis and *ex vivo* electrophysiology were performed in nicotine naïve female Wistar rats, and following sub-chronic nicotine exposure (0.36 mg/kg free base, 15 injections). Locomotor behavior was assessed at the first and last drug-exposure.

**Results:**

Acute exposure to nicotine *ex vivo* depresses excitatory neurotransmission by reducing the probability of transmitter release. Bath applied nicotine furthermore facilitated long-term synaptic depression induced by high frequency stimulation (HFS-LTD). The cannabinoid 1 receptor (CB1R) agonist WIN55,212-2 produced a robust synaptic depression of evoked potentials, and HFS-LTD was blocked by the CB1R antagonist AM251, suggesting that HFS-LTD in the female rat DLS is endocannabinoid mediated. Sub-chronic exposure to nicotine *in vivo* produced behavioral sensitization and electrophysiological recordings performed after 2-8 days abstinence revealed a sustained depression of evoked population spike amplitudes in the DLS, with no concomitant change in paired pulse ratio. Rats receiving sub-chronic nicotine exposure further demonstrated an increased neurophysiological responsiveness to nicotine with respect to both dopaminergic- and glutamatergic signaling. However, a tolerance towards the plasticity facilitating property of bath applied nicotine was developed during sub-chronic nicotine exposure *in vivo*. In addition, the dopamine D2 receptor agonist quinpirole selectively facilitate HFS-LTD in slices from nicotine naïve rats, suggesting that the tolerance may be associated with changes in dopaminergic signaling.

**Conclusion:**

Nicotine produces acute and sustained effects on striatal neurotransmission and synaptic plasticity in the female rat brain, which may contribute to the establishment of persistent nicotine taking habits.

## Introduction

Nicotine addiction is a major preventable risk factor for morbidity and mortality, yet few manage to maintain abstinent when exchanging cigarettes for nicotine replacement therapy (Livingstone-Banks et al., 2019). Defining underlying mechanism associated with nicotine addiction is thus pivotal to outline new strategies for successful nicotine cessation. Experimental studies have shown that drugs of abuse elicit glutamatergic synaptic plasticity that contributes to the reorganization of neural circuits, and putatively, the establishment of addictive behaviors (van Huijstee and Mansvelder, 2014; Christian et al., 2021; Pascoli et al., 2022). Dopamine, which is released in response to nicotine exposure, is a prerequisite for drug-induced plasticity (Belin et al., 2009; Lee et al., 2020; Rivera et al., 2021), and a key regulator of structural and synaptic plasticity (Urakubo et al., 2020; Speranza et al., 2021). In addition, nicotine activates nicotinic acetylcholine receptors (nAChRs), which also has been demonstrated to be important for synaptic plasticity at both inhibitory and excitatory synapses (Dani et al., 2001; Partridge et al., 2002; Adermark, 2011). By activating nAChRs and increasing dopamine levels, nicotine may thus facilitate the induction of synaptic plasticity mechanisms and elicit long-term neuroadaptations, which will outlast the presence of the drug.

Studies performed in male rodents have shown that nicotine especially facilitate synaptic plasticity in the form of endocannabinoid-mediated long-term depression (eCB-LTD; Adermark et al., 2019), and both nicotine self-administration as well as yoked nicotine exposure increase VTA dialysate levels of the cannabinoid 1 receptor (CB1R) agonist 2-arachidonoyl glycerol (2-AG; Buczynski et al., 2013). Antagonists targeting the CB1R furthermore blocks the motivational and dopamine-releasing effects of nicotine (Cohen et al., 2002; Cheer et al., 2007), and mice with a genetic deletion of the CB1R does not show conditioned place preference toward nicotine (Castane et al., 2002). The addictive properties of nicotine may in this extent be connected to facilitation of eCB signaling, which apart from its rewarding properties drive neuronal adaptations that may contribute to the formation of persistent drug-related habits and addictive behaviors (Hilario et al., 2007; Merritt et al., 2008; Hernandez et al., 2014; Bystrowska et al., 2019; Iyer et al., 2022). Repeated exposure to nicotine, however, has been shown to lead to decreased eCB levels in the striatum, without affecting CB1R expression (Gonzalez et al., 2002).

Importantly, smoking cigarettes is in part a gendered activity with sex-specific uptake trends and cessation patterns (Benowitz and Hatsukami, 1998). Smoking is more anxiolytic in women, and women are more likely to smoke in situations associated with stress or in response to negative affect (Perkins et al., 2012; Antin et al., 2017). Interestingly, human studies indicate that there is disparity with regards to nicotine-induced dopamine-release in women and men, where the reward system and ventral striatum is activated by nicotine in men, while dorsal striatal regions are recruited in women (Cosgrove et al., 2014). The dorsal striatum may thus be a key brain region of interest when assessing nicotine-induced neuroplasticity in females. Furthermore, animal studies indicate that female rats display higher tonic 2-AG signaling (Melis et al., 2013), which may affect the responsiveness to the putative nicotine-induced eCB release. Since previous studies outlining nicotine-induced facilitation of eCB signaling has been performed in male rats, the aim of this study was to study acute and sub-chronic effects by nicotine on neurotransmission and synaptic plasticity in the female rat brain. To this end, female Wistar rats were studied using *ex vivo* electrophysiology and *in vivo* microdialysis, and nicotine was administered through experimenter-controlled injections. Neurotransmission was monitored in the dorsolateral striatum, a brain region associated with the development of drug-related habits but also linked to neurophysiological responses to nicotine in women (Gerdeman et al., 2003; Belin et al., 2009; Cosgrove et al., 2014).

## Materials and methods

### Research outline

In the first set of experiments, acute effects displayed by nicotine on neurotransmission and long-term synaptic depression induced by high frequency stimulation (HFS-LTD) were assessed using *ex vivo* electrophysiology (*n* = 26 rats). In the next set of experiments, sustained neuroadaptations elicited by repeated nicotine exposure were outlined. Female rats received 15 experimenter-controlled nicotine injections (0.36 mg/kg, free base) distributed over 3 weeks and locomotor activity was measured at the first and last exposure (*n* = 30). Baseline dopamine levels and dopamine release evoked by nicotine were recorded in the DLS using *in vivo* microdialysis, while synaptic activity and HFS-LTD were monitored by *ex vivo* electrophysiology.

### Animals

Female Wistar rats (Envigo, Netherlands) were group housed (3/cage) and kept on a 12/12-h light/dark cycle at 22°C with 50% humidity with free access to food and water. All experiments were approved by the Gothenburg Animal Research Ethics Committee and conducted during the light time cycle. Estrous cycle was estimated by visual assessment of vaginal smear at the time point for neurophysiological assessments.

### Electrophysiological recordings

#### Brain slice preparation

**Figure 1:**
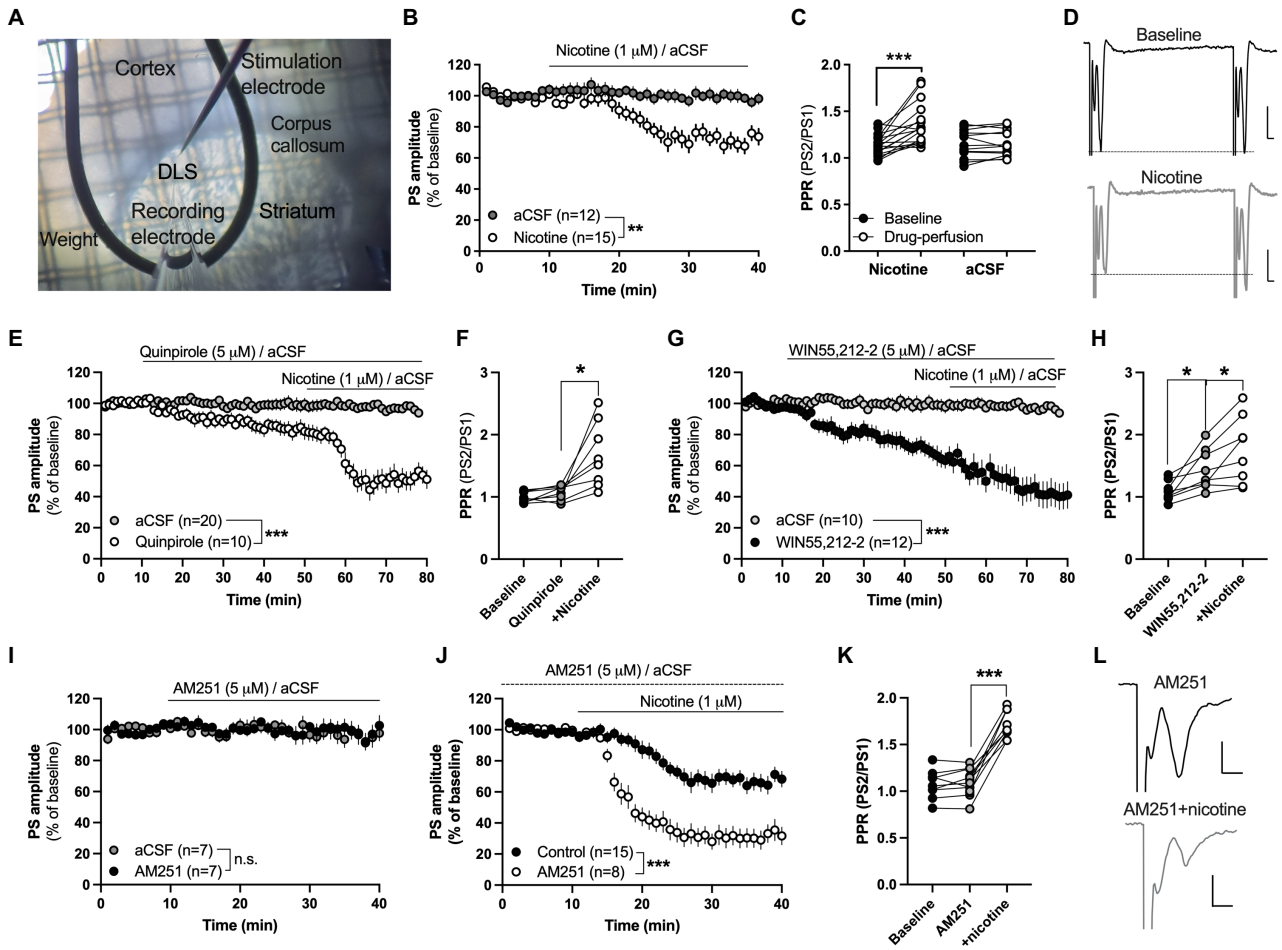
Acute nicotine exposure depresses evoked potentials and promotes HFS-LTD in the female rat striatum. **(A)** Micrograph showing the position of recording and stimulation electrode in the DLS. Recording and stimulating electrodes were positioned close to the corpus callosum in the lateral part of the dorsal striatum. Note the visual texture of the striatum and the dark shade of the corpus callosum which clearly marks the transition to the cortex. **(B)** Evoked field potentials were significantly depressed by bath perfusion of nicotine (1 μM). **(C)** Nicotine perfusion significantly increased PPR. **(D)** Example traces showing field potentials evoked with a paired pulse stimulation protocol delivered with a 50 ms interpulse interval at baseline (upper trace) and after 25 min nicotine perfusion (lower trace). Note that the first pulse decreases to a greater extent in response to nicotine perfusion, thereby resulting in increased PPR. Calibration: 0.2 mV, 2 ms. **(E,F)** The dopamine D2 receptor agonist quinpirole (5 μM) slightly depressed PS amplitude but did not occlude nicotine-induced synaptic depression or the increase in PPR. **(G,H)** Bath perfusion of the CB1R agonist WIN55,212-2 (3 μM) significantly depressed PS amplitude and increased PPR, and nicotine co-perfusion further potentiated synaptic depression. **(I)** The CB1R antagonist AM251 (3 μM) did not modulate PS amplitude by itself. **(J)** Synaptic depression induced by acute nicotine perfusion was significantly enhanced in AM251-pretreated slices. **(K)** PPR was not modulated by AM251, but robustly enhanced when nicotine was co-administered. **(L)** Example traces showing evoked PS amplitude at AM251-treated baseline (upper trace) and following nicotine co-perfusion (lower trace). Not that the pre pulse is not affected during nicotine co-perfusion demonstrating that nicotine affects synaptic transmission. Data are mean values ± SEM. *n* = number of slices, taken from at least three animals. *p < 0.05, ***p < 0.001.

Electrophysiological recordings were conducted in brain slices from naïve rats (10–14 weeks old, Figure 1) and animals receiving sub-chronic treatment (15 injections over 3 weeks) of either nicotine or saline (approximately 4 months old). In brief, animals were anesthetized with isoflurane and the brain was quickly removed and submerged in ice-cold modified aCSF containing (in mm): 220 sucrose, 2 KCl, 6 MgCl_2_, 26 NaHCO_3_, 1.3 NaH_2_PO_4_, 0.2 CaCl_2_, and 10 d-glucose, continuously bubbled with a gas mixture of 95% O_2_/5% CO_2_. Brains were sectioned coronally at 250 μm using a VT 1200S Vibratome (Leica Microsystems, Bromma, Sweden). The slices were transferred to a custom-made incubation chamber with conventional aCSF containing (in mm): 124 NaCl, 4.5 KCl, 2 CaCl_2_, 1 MgCl_2_, 26 NaHCO_3_, 1.2 NaH_2_PO_4_, and 10 d-glucose, with osmolarity adjusted to 315–320 mOsm with sucrose, and continuously bubbled with 95% O_2_/5% CO_2_. Slices were incubated in aCSF for 30 min at 30°C and at room temperature for the remainder of the day.

#### Field potential recordings

**Figure 2:**
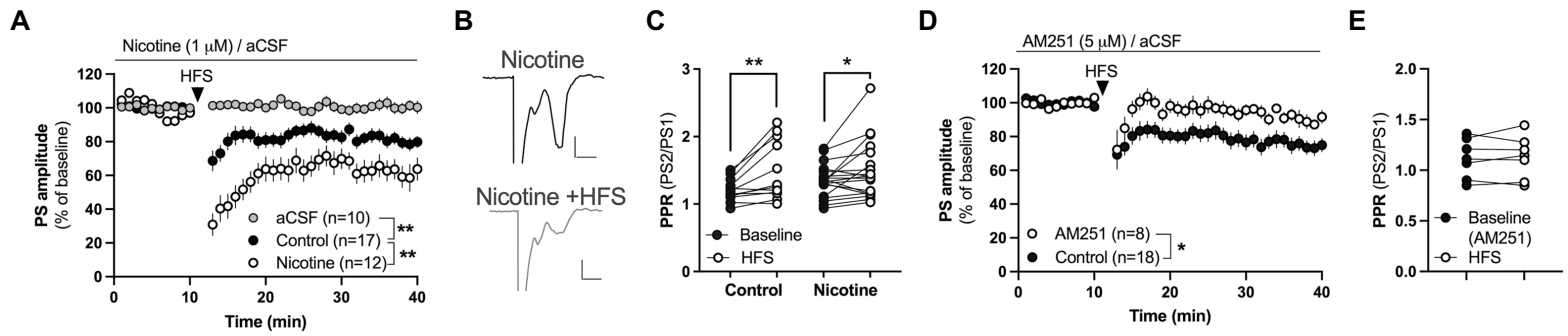
Nicotine facilitates endocannabinoid-mediated LTD in the DLS. **(A)** HFS produced LTD in both aCSF-treated control slices and in nicotine pre-treated slices, but synaptic depression was significantly enhanced by nicotine. **(B)** Example traces showing evoked potentials at nicotine-treated baseline (upper trace) and 25 min after HFS (lower trace). Calibration: 0.2 mV, 2 ms. **(C)** HFS increased PPR in both aCSF-treated control slices and slices treated with nicotine, indicating that HFS decreases the probability of transmitter release. **(D,E)** HFS did not induce LTD in brain slices-pre-treated with AM251. Data are mean values ± SEM. *n* = number of slices, taken from at least three animals. *p < 0.05, **p < 0.01.

Field potential recordings were performed as previously described (Adermark et al., 2011a). In brief, one hemisphere of the slice was perfused with prewarmed aCSF (30°C) and field population spikes (PSs) were evoked with a stimulation electrode positioned dorsal in close vicinity (0.2–0.3 mm) to the recording electrode (World Precision Instruments, FL, United States; Figure 2A). Striatal field potential recordings primarily reflect AMPA-receptor activation (Lagstrom et al., 2019), and the experiments were performed in order to outline the effects displayed by acute and repeated nicotine exposure on striatal neurotransmission and stimulation-induced plasticity. Changes in release probability was measured by employing a paired pulse stimulation protocol with a 50 ms interpulse interval. Paired pulse stimulation was evoked at 0.1 Hz, and paired pulse ratio (PPR) was calculated by dividing the second PS amplitude with the first evoked response.

In the first set of experiments the acute effect displayed by nicotine on striatal neurotransmission was determined. Response amplitudes were set to approximately 60% of the maximal response, and a stable baseline was recorded for at least 10 min before drugs were administered through bath perfusion. Nicotine was perfused at a concentration of 1 μM, which has previously been shown to depress the frequency of glutamatergic inputs onto striatal MSNs in brain slices from male rats (Licheri et al., 2018). To determine putative signaling pathways activated by nicotine, slices were pre-treated with the dopamine D2 receptor agonist quinpirole (dissolved in H_2_O to 50 mM and used at 5 μM), the CB1R agonist WIN55,212-2 (dissolved in DMSO to 20 mM and used at 3 μM) or the CB1R antagonist AM251 (dissolved in DMSO to 20 mM and used at 3 μM).

In the next set of recordings, the ability for nicotine to facilitate synaptic plasticity in the form of long-term depression (LTD) was assessed by administering four trains of 100 pulses delivered at 100 Hz (HFS). This protocol has previously been shown to elicit robust eCB-mediated LTD in brain slices from male rats (HFS-LTD; Adermark and Lovinger, 2009). To further determine if CB1R signaling may underly HFS-LTD also in brain slices from female rats, slices were pre-treated with the CB1R antagonist AM251 (3 μM) prior to HFS.

In the last set of experiments, sustained effects elicited by repeated nicotine exposure on neurotransmission and stimulation-induced plasticity were determined. To this end, stimulation/response curves were evoked with increasing stimulation intensity (12–48 μA) in slices from rats receiving either sub-chronic nicotine exposure or vehicle injections. Changes in the responsiveness to acute nicotine perfusion and HFS-LTD were also assessed. eCB-LTD in male rats requires dopamine D2 signaling (Kreitzer and Malenka, 2005), and nicotine has been suggested to facilitate eCB-LTD by stimulating dopamine D2 receptor activation (Adermark et al., 2019). In a subset of recordings brain slices were thus perfused with the dopamine D2 receptor agonist quinpirole (5 μM) prior to HFS-stimulation. Signals were amplified with a custom-made amplifier, filtered at 3 kHz, and digitized at 8 kHz.

### Nicotine-treatment and behavioral sensitization

Rats (10–12 weeks old) were randomly assigned to receive either nicotine (0.36 mg/kg, free base, pH normalized with NaHCO_3_) or saline (0.9% NaCl) over a period of 3 weeks. Subcutaneous injections of nicotine or saline were given in a discontinuous manner over 3 weeks (15 injections in total). This treatment protocol has previously been shown to induce a long-lasting behavioral sensitization to nicotine that sustains for at least 7 months in male rats (Morud et al., 2016).

A behavioral sensitization toward the locomotor stimulatory properties of psychostimulants with repeated drug-exposure is well established in animal models and is believed to be associated with aberrant dopamine signaling (Jackson and Moghaddam, 2001; Phillips et al., 2003; Rouillon et al., 2008; Jones et al., 2010). As a proxy to establish that repeated nicotine exposure had elicit behavioral and neurophysiological transformations locomotor behavior was monitored in a subset of rats (nine vehicle, 10 nicotine) during the first and last (15th) injection to either vehicle or nicotine. Vertical and horizontal movements were registered in an open-field arena (Med Assoc., Fairfax, VT, United States) placed in a sound attenuated, ventilated, and dim lit box (Adermark et al., 2015). The open-field arena was equipped with a two-layer grid, consisting of rows of photocell beams, and consecutive beam breaks were tracked by a computer-based system (Activity Monitor 7, Med Assoc., St. Albans, VT, United States). Rats were allowed to habituate to the box for 30 min and were then injected with nicotine (0.36 mg/kg, s.c.) or vehicle (0.9% NaCl), after which locomotor behavior was registered for another 30 min. Data was registered to determine if the locomotor stimulatory property of nicotine would increase with repeated nicotine administration, and if repeated nicotine exposure would lead to changes in locomotion also during the drug-free habituation period.

### Microdialysis

Surgery for *in vivo* microdialysis was performed 3 days after the last nicotine injection and the experiments were performed after a total of 5 days abstinence. Microdialysis probe placement was performed as previously described (Adermark et al., 2022). In brief, a custom-made I-shaped dialysis probe was lowered into the DLS [A/P: +1.2, M/L: −3.5 relative to bregma, D/V: −5.5 relative to dura mater (Paxinos and Watson, 2007)] and fixed to the scull, together with the anchoring screws, using Harvard cement (DAB Dental AB, Gothenburg, Sweden). Rats were allowed to recover for 48 h before initiation of the *in vivo* microdialysis experiment.

Microdialysis experiments were performed in awake and freely moving animals [*n* = 19, weight 230–250 g (approximately 4 months old)]. In brief, the dialysis probe was perfused with Ringer’s solution at a rate of 2 μl/min and dialysate samples were collected every 20 min. When four stable dopamine baseline samples (less than ±10% fluctuation) had been retrieved, nicotine (0.36 mg/kg) was administered subcutaneously. The dialysate samples were analyzed online using high performance liquid chromatography (HPLC) with electrochemical detection as described previously (Ulenius et al., 2020). After the experiment, brains were removed, fixed in formaline-free fixative (Accustain, Sigma-Aldrich, Stockholm, Sweden) and stored (+4°C) for 4–7 days until verification of probe placement. One rat was excluded for incorrect probe placement.

### Statistical analysis

Electrophysiological data were extracted using Clampfit 10.2 and Microsoft excel. All data were assembled and analyzed using GraphPad Prism 9 (GraphPad Software, San Diego, CA, United States). Two-way analysis of variance (ANOVA) was used for comparisons over time, and input/output function, while paired or unpaired t tests were used when applicable. All parameters are given as mean ± standard error of the mean (SEM), and the level of significance was set to *p* < 0.05.

## Results

### Acute nicotine perfusion depresses evoked field potentials in the female rat striatum

In the first set of experiments, the effect displayed by acute nicotine exposure on striatal neurotransmission was assessed by electrophysiological field potential recordings in brain slices from nicotine-naïve rats. Bath perfusion of nicotine (1 μM) depressed evoked field potentials and increased PPR indicating that nicotine reduces the probability of transmitter release (PS amplitude: Repeated measures ANOVA: main effect treatment_(nicotine acute)_: *F*_(1,25)_ = 13.68, *p* = 0.0011; time: *F*_(27,625)_ = 15.88, *p* < 0.001; time × treatment: *F*_(27,625)_ = 7.716, *p* < 0.001; PPR_(nicotine acute)_: Student’s *t* test: *t*_(16)_ = 4.408, *p* < 0.001; Figures 1B,C). aCSF perfusion alone did not affect PPR (*t*_(12)_ = 0.7358, *p* = 0.4760; Figure 1C).

Nicotine increases dopaminergic neurotransmission and the dopamine D2 receptor agonist quinpirole has been shown to occlude nicotine-induced synaptic depression in brain slices from male rats (Licheri et al., 2018). Quinpirole (5 μM) depressed PS amplitude compared to aCSF-treated control (main effect treatment_(quinpirole acute)_: *F*_(1,28)_ = 8.537, *p* = 0.0068; time: *F*_(35,980)_ = 3.060, *p* < 0.001; time × treatment: *F*_(35,980)_ = 1.938, *p* = 0.0010; Figure 1E), with a trend toward increased PPR following 40 min quinpirole perfusion (*t*_(7)_ = 2.147, *p* = 0.0689; Figure 1F). Pre-treatment with quinpirole, however, did not prevent nicotine-induced depression (main effect treatment_(nicotine acute)_: *F*_(1,16)_ = 27.18, *p* < 0.001; time: *F*_(25,400)_ = 6.619 *p* < 0.001; time × treatment: *F*_(25,400)_ = 5.796, *p* < 0.001) or the nicotine-induced increase in PPR (stats; PPR: *t*_(7)_ = 3.473, *p* = 0.0104; Figures 1E,F).

Nicotine has also been postulated to stimulate eCB signaling and might thus depress PS amplitude *via* activation of presynaptic CB1Rs. Perfusion of the CB1R agonist WIN55,212-2 (3 μM) depressed PS amplitude (main effect treatment_(WIN55,212-2 acute)_: *F*_(1,20)_ = 16.37, *p* < 0.001; time: *F*_(35,700)_ = 7.295, *p* < 0.001; time × treatment: *F*_(35,700)_ = 4.992, *p* < 0.0001) and increased PPR by itself (PPR: *t*_(7)_ = 3.473, *p* = 0.0104; Figures 1G,H). Co-perfusion with nicotine resulted in a continuous depression of PS amplitude (main effect treatment_(nicotine acute)_: *F*_(1,14)_ = 24.37, *p* < 0.001; time: *F*_(25,350)_ = 5.513, *p* < 0.001; time × treatment: *F*_(25, 350)_ = 4.510, *p* < 0.0001) and a further increase in PPR (*t*_(7)_ = 3.019, *p* = 0.0194; Figures 1G,H). Since the baseline was steadily decreasing by WIN55,212-2-perfusion alone, another experiment was performed where slices were pre-treated with the CB1R antagonist AM251 (3 μM) prior to nicotine. AM251 did not affect PS amplitude or PPR by itself (AM251 vs. aCSF: treatment: *F*_(1,12)_ = 0.1056, *p* = 0.7508; time: *F*_(25,300)_ = 0.7813, *p* = 0.7655; time × drug: *F*_(25,300)_ = 0.6216, *p* = 0.9228; PPR: *t*_(8)_ = 1.083, *p* = 0.3105; Figures 1I,K). Co-administration with nicotine, however, demonstrated an enhanced synaptic depression by nicotine when compared to control slices (main effect treatment_(nicotine acute)_: *F*_(1,18)_ = 28.70, *p* < 0.001; time: *F*_(25,450)_ = 34.88, *p* < 0.001; time × drug: *F*_(25,450)_ = 2.336, *p* < 0.001), and a robust increase in PPR (*t*_(8)_ = 11.12, *p* < 0.001; Figures 1J,K).

### Nicotine promotes synaptic plasticity in the form of endocannabinoid-mediated LTD

In the next set of experiments the ability for nicotine to promote HFS-LTD was assessed. HFS produced a robust synaptic depression of PS amplitude in control slices that was concomitant with an increase in PPR indicative of a reduced probability of transmitter release [*F*_(1,30)_ = 11.16, *p* = 0.0022; time: *F*_(25,750)_ = 8.134, *p* < 0.001; time × drug: *F*_(25,750)_ = 1.067, *p* = 0.3752; PPR (*t*_(11)_ = 3.324, *p* = 0.0068; Figures 2A,C)]. While HFS-LTD in DLS has been suggested to be mediated by eCB-signaling in male rats (Gerdeman and Lovinger, 2001), CB1R expression has been shown to be lower in the female mouse striatum compared to males (Liu et al., 2020). To determine if HFS-LTD was associated with eCB signaling also in the female rat striatum, slices were bath perfused with the CB1R antagonist AM251 (3 μM). AM251 blocked synaptic depression mediated by HFS (main effect treatment_(AM251 vs. control)_: *F*_(1,24)_ = 6.630, *p* = 0.0166; time: *F*_(25,600)_ = 2.482, *p* < 0.001; time × drug: *F*_(25,600)_ = 0.4267, *p* = 0.9940; Figure 2D), and the increase in PPR (*t*_(6)_ = 0.3975, *p* = 0.7048; Figure 2E). Importantly, pre-treatment for 30 min with bath applied nicotine (1 μM) significantly enhanced HFS-LTD (aCSF vs. nicotine acute: treatment: *F*_(1,31)_ = 2.60, *p* = 0.0013; time: *F*_(27,837)_ = 8.134, *p* < 0.001; time × drug: *F*_(27,837)_ = 2.794, *p* < 0.001; PPR_(nicotine acute + HFS)_: *t*_(18)_ = 2.267, *p* = 0.03609), suggesting that acute nicotine exposure facilitates HFS-LTD also in the DLS of female rats (Figures 2A–C).

### Repeated nicotine exposure produces robust behavioral sensitization in female rats

**Figure 3:**
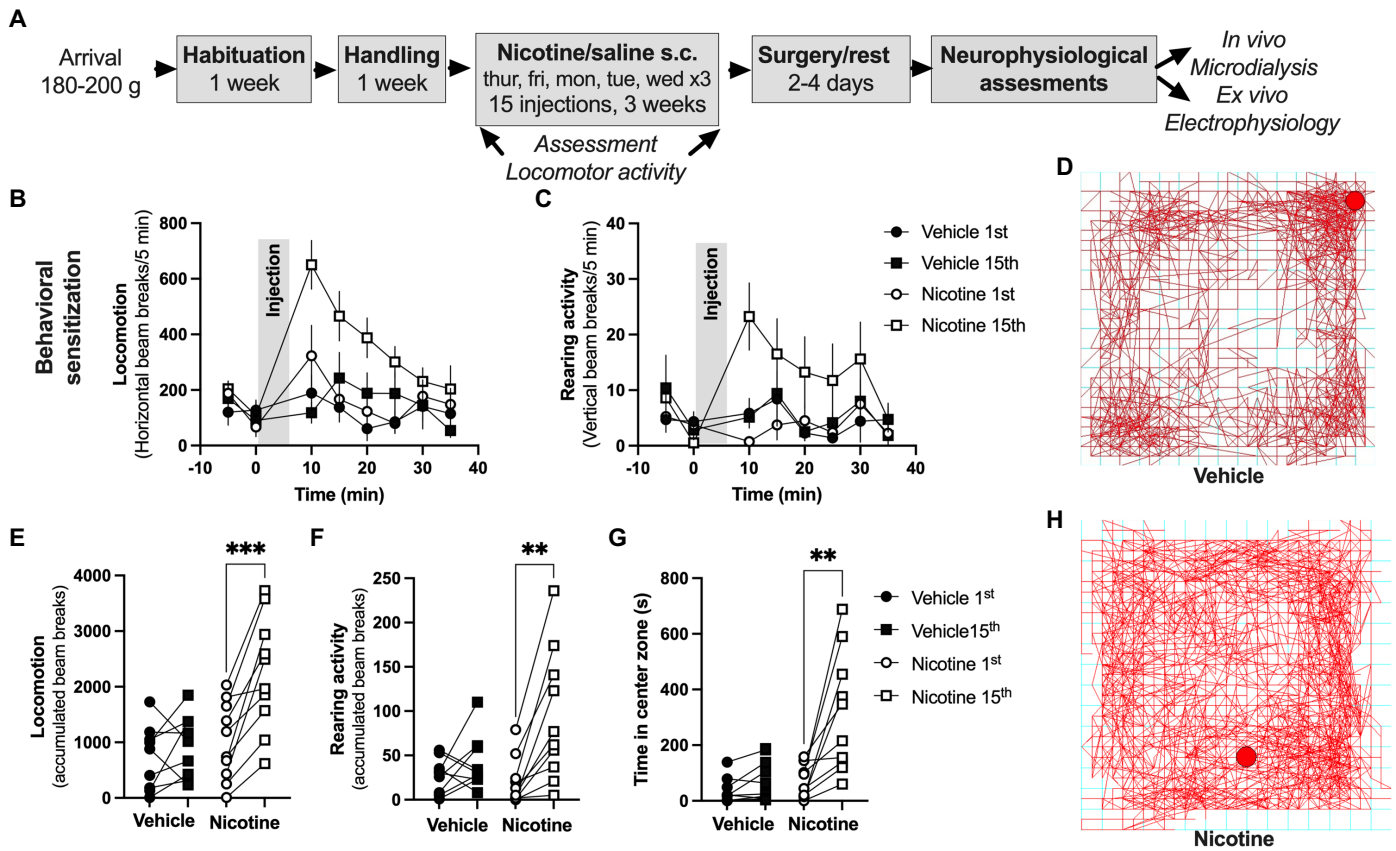
Repeated nicotine exposure produces behavioral sensitization in female rats. **(A)** Timeline for the described experiments. **(B,C)** Time-course graphs showing locomotor activity and rearing activity in response to nicotine injection. **(D)** Example picture showing ambulatory movement in a rat receiving vehicle injection. **(E,F)** Accumulated beam breaks elicited by either saline or nicotine exposure during the first and last injection demonstrated a selective increase in locomotion in rats receiving repeated nicotine injections. **(G)** Time spent in center zone was increased in response to repeated nicotine injections. **(H)** Example picture showing ambulatory movement in a rat receiving nicotine injection. Data are mean values ± SEM and based on 9–10 rats/treatment-group. *p < 0.05, **p < 0.01.

Acute administration depressed evoked potentials in the DLS and facilitated LTD. In a way to outline if repeated nicotine exposure would lead to sustained neuroadaptations, female rats underwent sub-chronic nicotine exposure through experimenter-controlled nicotine injections (0.36 mg/kg, s.c., total of 15 injections) delivered over a period of 3 weeks. As a proxy for behavioral and neurophysiological transformations, nicotine-induced behavioral sensitization was monitored in locomotion boxes. Repeated nicotine exposure produced robust behavioral sensitization demonstrated as increased locomotor activity in response to a nicotine challenge following sub-chronic nicotine treatment (paired *t*-test, 1st vs. 15th injection: horizontal beam breaks: vehicle: *t*_(8)_ = 0.9802, *p* = 0.3557; nicotine: *t*_(9)_ = 5.325, *p* < 0.001; vertical beam breaks: vehicle: *t*_(8)_ = 1.177, *p* = 0.2729; nicotine: *t*_(9)_ = 4.223, *p* = 0.0022; Figures 3B–F). In addition, following drug-administration, an increase in the time spent in center zone was seen selectively in rats receiving repeated nicotine exposures (paired *t*-test, 1st vs. 15th injection vehicle: *t*_(8)_ = 1.884, *p* = 0.0962; nicotine: *t*_(9)_ = 3.752, *p* = 0.0045; Figures 3G,H). Neither time spent in center zone (vehicle 15th vs. nicotine 15th: *t*_(13)_ = 0.4298, *p* = 0.6744) nor baseline locomotion (vehicle 15th vs. nicotine 15th: *t*_(13)_ = 0.1657, *p* = 0.8709) were affected by sub-chronic nicotine administration when monitored during the habituation to the locomotor box (first 30 min, prior to drug-administration), suggesting that locomotor behavior during drug-free conditions were not significantly altered.

### Neurophysiological transformations following sub-chronic nicotine exposure

**Figure 4:**
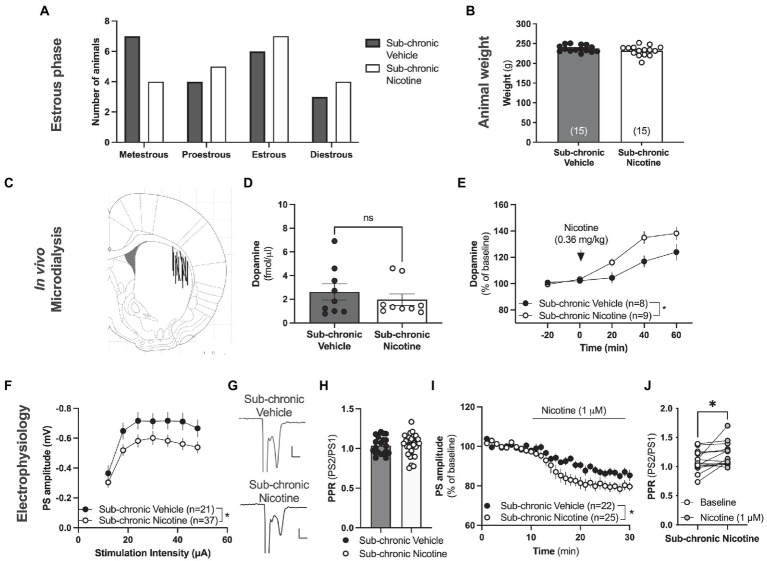
Neurophysiological transformations following sub-chronic nicotine exposure. **(A)** Estimated estrus phase based on vaginal smears for animals used during neurophysiological assessments. **(B)** Animal weight did not differ between treatment groups at the time point for neurophysiological assessments. **(C)** Schematic drawing showing the position of the active space of the microdialysis probe. **(D)** The microdialysate level of dopamine was not significantly altered by sub-chronic nicotine exposure. **(E)** Acute administration of nicotine, as indicated by the arrow, increased striatal dopamine to a significantly greater extent in animals receiving sub-chronic nicotine exposure. **(F)** *Ex vivo* electrophysiological recordings demonstrated that the amplitude of evoked potentials was significantly depressed in the DLS of animals previously receiving nicotine. **(G)** Example traces showing evoked PSs in a brain slice from a vehicle-treated rat (upper trace) and a rat receiving sub-chronic nicotine exposure (lower trace). **(H)** PPR was not significantly modulated by sub-chronic nicotine exposure. **(I)** Bath perfused nicotine depressed evoked potentials to a significantly greater extent in brain slices from rats receiving sub-chronic nicotine exposure. **(J)** Synaptic depression induced by acute nicotine exposure was accompanied by an increase in PPR, indicating that nicotine reduces the probability of transmitter release. Data are mean values ± SEM. *n* = number of animals in **(B,E)**, and number of slices in **(F,I)**. Slices were taken from at least five animals/treatment. *p < 0.05, **p < 0.01.

In the next step, neurophysiological assessments were conducted *in vivo* and *ex vivo* in animals previously receiving sub-chronic administration of either saline or nicotine. Estimation of estrus phase did not reveal any significant deviations between treatment groups (Chi square test: *p* = 0.7652; Figure 4A). There was also no effect by repeated nicotine exposure on weight (*t*_(16)_ = 0.7734, *p* = 0.4506; Figure 4B).

*In vivo* microdialysis performed in the DLS 5 days after the last drug-treatment demonstrated no change in baseline extracellular dopamine levels in the DLS following sub-chronic nicotine exposure (*t*_(28)_ = 1.439, *p* = 0.1614; Figure 4D). The relative responsiveness to acute nicotine administration (0.36 mg/kg), however, was significantly enhanced in animals previously receiving sub-chronic nicotine exposure (sub-chronic nicotine vs. vehicle: treatment: *F*_(1,16)_ = 6.431, *p* = 0.0220; time: *F*_(2,32)_ = 36.93, *p* < 0.001; time × treatment: *F*_(2,32)_ = 0.8087, *p* = 0.4543; Figure 4E), indicative of a sensitized response to nicotine with regards to the relative dopamine release.

Electrophysiological recordings performed on nicotine abstinent rats 2–7 days after the last nicotine exposure demonstrated a sustained synaptic depression of evoked potentials in brain slices from rats receiving sub-chronic nicotine treatment (treatment: *F*_(1,56)_ = 4.179, *p* = 0.00456; time: *F*_(6,336)_ = 52.47, *p* < 0.001; time × drug: *F*_(6,336)_ = 0.8307, *p* = 0.5468; Figures 4F,G), with no concomitant change in PPR (*t*_(49)_ = 0.28, *p* = 0.7806; Figure 4H), indicating that repeated nicotine exposure may lead to sustained postsynaptic transformations that outlasts the presence of the drug. In addition, bath perfused nicotine (1 μM) further depressed PS amplitude to a significantly greater extent in brain slices from animals previously receiving nicotine (sub-chronic nicotine vs. vehicle: treatment: *F*_(1,45)_ = 8.898, *p* = 0.0046; time: *F*_(15,675)_ = 0.878, *p* < 0.001; time × treatment: *F*_(15,675)_ = 0.818, *p* = 0.659; Figure 4I), indicating a sensitized response to nicotine also with respect to glutamatergic neurotransmission. Bath perfused nicotine increased PPR also in slices from rats receiving sub-chronic nicotine exposure (stats *t*_(14)_ = 2.501, *p* = 0.0254; Figure 4J).

### Regulation of synaptic plasticity in the form of HFS-LTD is modulated by repeated nicotine exposure in the female rat brain

**Figure 5:**
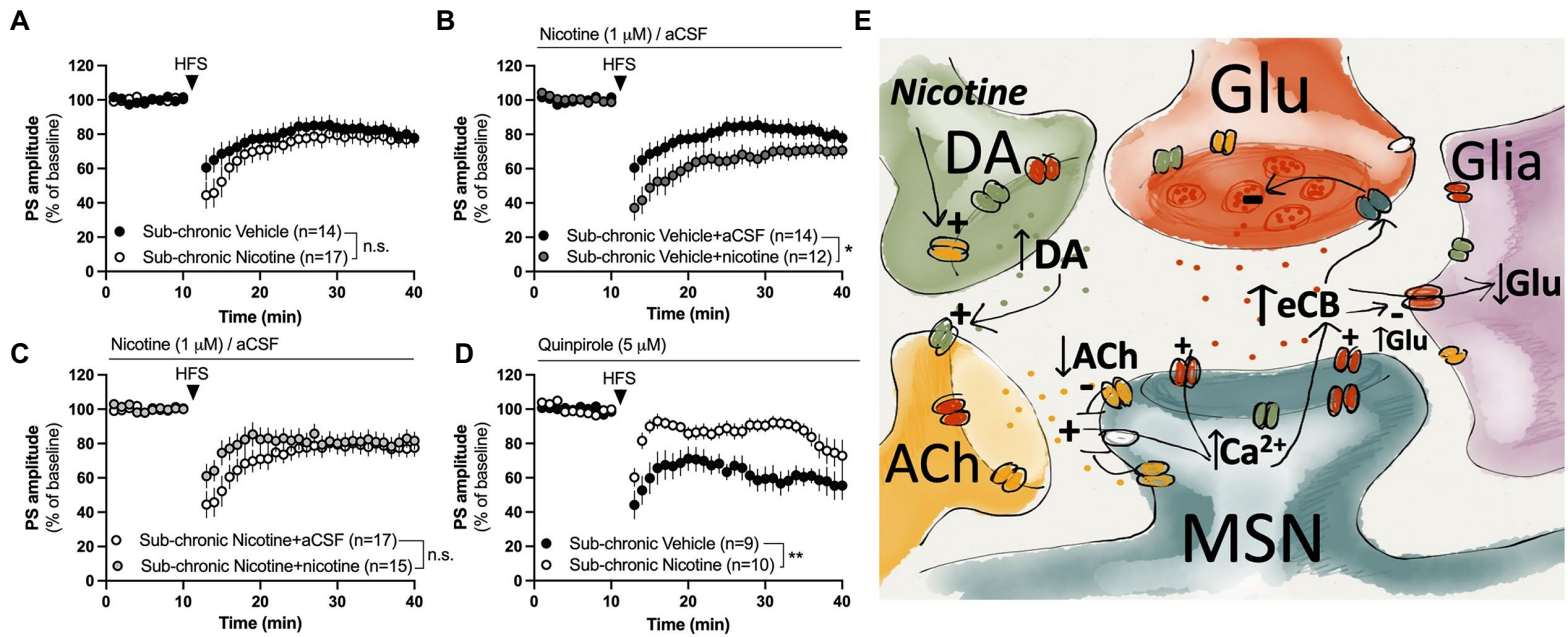
Nicotine or dopamine D2 receptor activation does not facilitate LTD in slices from female rats receiving sub-chronic nicotine treatment. **(A)** HFS-LTD was not significantly modulated during acute withdrawal (2–7 days) after the last nicotine exposure. **(B,C)** Bath perfusion of nicotine facilitated HFS-LTD in slices from vehicle-treated rats but not from nicotine-treated rats. **(D)** The dopamine D2 receptor agonist quinpirole selectively facilitated HFS-LTD in slices from vehicle-treated rats. **(E)** Simplified schematic drawing depicting putative signaling pathways that nicotine may recruit to modulate neurotransmission and HFS-LTD in the DLS. Acute administration of nicotine may weakly stimulate eCB production and release by activating nAChRs located on dopaminergic terminals, thereby increasing the inhibitory tone by dopamine on cholinergic neurons. Reduced cholinergic tone relieves the inhibition of L-type calcium channels (Wang et al., 2006), thereby facilitating calcium influx and eCB production in MSNs. When nicotine is combined with HFS, eCB signaling is potentiated compared to HFS alone, thereby leading to a more robust depression of PS amplitude. Following sub-chronic nicotine exposure, however, the regulatory role of dopamine D2 receptors appears to be altered. Endocannabinoids may also reduce glutamate uptake (Brown et al., 2003), thereby increasing extrasynaptic glutamate levels and further promoting eCB signaling and synaptic depression. Data are mean values ± SEM. *n* = number of slices, taken from at least three animals. *p < 0.05, **p < 0.01.

In the last sets of experiments, the influence displayed by sub-chronic nicotine exposure *in vivo* on the induction and expression of striatal long-term synaptic plasticity was outlined. While eCB levels have been reported to be depressed following repeated nicotine exposure in male rats (Gonzalez et al., 2002), HFS-LTD was not impaired in brain slices from rats receiving sub-chronic nicotine exposure (sub-chronic nicotine vs. vehicle: treatment: *F*_(1,31)_ = 0.7419, *p* = 0.3957; time: *F*_(27,837)_ = 19.68, *p* < 0.001; time × treatment: *F*_(27,837)_ = 1.396, *p* = 0.0878; Figure 5A). However, bath perfused nicotine (1 μM) facilitated HFS-LTD in brain slices from vehicle treated rats (treatment_(nicotine acute)_: *F*_(1,25)_ = 4.678, *p* = 0.0403; time: *F*_(27,675)_ = 18.89, *p* < 0.001; time × treatment: *F*_(27,675)_ = 1.480, *p* = 0.0566; Figure 5B), but not in brain slices from rats receiving sub-chronic nicotine treatment (treatment_(nicotine acute)_: *F*_(1,30)_ = 0.1912, *p* = 0.6651; time: *F*_(27,810)_ = 13.74, *p* < 0.001; time × treatment: *F*_(27, 810)_ = 2.306, *p* = 0.0002; Figure 5C).

eCB-LTD in male rats requires dopamine D2 signaling (Kreitzer and Malenka, 2005), and nicotine has been suggested to facilitate eCB-LTD by stimulating dopamine D2 receptor activation (Adermark et al., 2019). Previous studies performed on brain slices from male rats has suggested that nicotine produces changes in dopamine D2 receptor signaling which leads to distorted synaptic plasticity mechanisms (Adermark et al., 2019). In the last set of experiments responsiveness to dopamine D2 receptor activation and effects on HFS-LTD were assessed in brain slices from female rats receiving either sub-chronic treatment with nicotine or vehicle. Administration of the dopamine D2 receptor agonist quinpirole slightly depressed PS amplitude to a similar extent in both treatment groups (vehicle vs. sub-chronic nicotine: treatment: *F*_(1,20)_ = 0.7264, *p* = 0.4042; time: *F*_(15,300)_ = 7.064, *p* < 0.001; time × treatment: *F*_(15,300)_ = 2.348, *p* = 0.0034; data not shown). Pre-treatment with quinpirole selectively enhanced HFS-LTD in brain slices from vehicle-treated rats (vehicle aCSF vs. vehicle quinpirole: treatment: *F*_(1,21)_ = 4.343, *p* = 0.04961; time: *F*_(27,567)_ = 8.182, *p* < 0.001; time × treatment: *F*_(27,567)_ = 4.297, *p* < 0.001), and HFS-LTD in quinpirole-treated brain slices was significantly reduced in slices from rats receiving sub-chronic nicotine-treatment compared to slices from vehicle-treated control (vehicle vs. sub-chronic nicotine: treatment: *F*_(1,17)_ = 11.87, *p* = 0.0031; time: *F*_(27,459)_ = 7.035, *p* < 0.001; time × treatment: *F*_(27,459)_ = 1.758, *p* = 0.0116; Figure 5D).

## Discussion

The data presented here shows that nicotine acutely depresses neurotransmission in a manner that is not driven by CB1R activation or dopamine D2 receptor activation, indicating that dopamine may regulate these acute effects in a partially sex-dependent manner (Licheri et al., 2018). In addition, HFS produced eCB mediated long-standing synaptic plasticity in the DLS of female rats, and nicotine pre-exposure significantly enhanced this form of LTD. This finding is in agreement with previous data retrieved from male rats (Adermark et al., 2019). We also show that repeated exposure to nicotine produces a sustained depression of evoked potentials in the DLS and give rise to a sensitized response to nicotine with regards to both locomotor behavior, as well as dopaminergic and glutamatergic signaling in the DLS. At the same time, our data show that the plasticity facilitating property of nicotine vanishes following repeated nicotine exposure. We also demonstrate that dopamine D2 receptor activation is insufficient to facilitate HFS-LTD in slices from rats previously receiving nicotine. These findings suggest that repeated nicotine exposure produces neuroplastic transformations in the DLS and impaired regulation of stimulation-induced long-term depression, which in the extension may reduce behavioral flexibility and contribute to the development of persistent habits.

Acute administration of nicotine depressed PS amplitude in a manner that was concomitant with a decrease in the probability of transmitter release. This finding is in agreement with previous studies performed on male rats, showing that nicotine acutely depresses the frequency but not amplitude of spontaneous excitatory post synaptic currents in medium spiny neurons in the DLS (Licheri et al., 2018). The exact mechanisms underlying nicotine-induced neuroplasticity in the striatum is not fully understood, but dopamine D2 receptor signaling appears to mediate the down-stream effects of nAChR activation (Licheri et al., 2018; Grieder et al., 2022). Dopamine D2 receptors are also critical for induction of nicotine-induced conditioned place preference (CPP) in mice (Wilar et al., 2019), and the improving effect of nicotine on stress-induced memory impairment (Keshavarzian et al., 2018). Increased levels of dopamine D2 receptor mRNA and receptor binding has also been reported following repeated nicotine administration in experimental studies (Metaxas et al., 2010; Adermark et al., 2016), while a decrease in DAT availability is associated with tobacco addiction in humans (Leroy et al., 2012). Changes in dopaminergic neurotransmission may thus be involved in mediating both acute and protracted neuroplasticity elicited by nicotine. However, while data from male rats has shown that nicotine-induced synaptic depression is occluded by dopamine D2 receptor activation and blocked by dopamine D2 receptor antagonist (Licheri et al., 2018), this was not supported by the data presented here. While the net effect by nicotine on striatal neurotransmission appears to be the same, the mechanisms underlying synaptic depression may thus be partially sex-dependent. Interestingly, while HFS-LTD was enhanced by nicotine, synaptic depression elicited by nicotine alone was potentiated by CB1R antagonist. Since low levels of eCBs has been proposed to act primarily on GABAergic terminals (Uchigashima et al., 2007; Adermark and Lovinger, 2009), it is possible that nicotine stimulates eCB release but that the amount released is insufficient to affect glutamatergic synapses. When combined with HFS, however, eCB-levels are high enough to act on both excitatory and inhibitory terminals (Adermark and Lovinger, 2009). Importantly, the data presented here shows that nicotine-induced synaptic depression engage several signaling pathways and more research is required to fully understand the mechanisms involved.

HFS induced a long-term depression of PS amplitude with a concomitant increase in PPR indicating that synaptic depression is expressed presynaptically. While eCB-signaling has been shown to be important for HFS-LTD in the DLS of male rats (Gerdeman and Lovinger, 2001; Kreitzer and Malenka, 2005; Adermark and Lovinger, 2009), CB1R expression has been shown to be lower in the female mouse striatum compared to males (Liu et al., 2020), and other signaling pathways may thus be recruited. However, HFS-LTD was blocked in slices pre-treated with the CB1R antagonist AM251, showing that eCB-signaling is important for LTD also in the DLS of female rats. Endocannabinoids are produced on demand and the indiction of eCB signaling involves a complex interplay of dopaminergic, glutamatergic and cholinergic receptors (Kreitzer and Malenka, 2005; Wang et al., 2006; Adermark, 2011; Liput et al., 2022). Direct activation of L-type calcium channels produces eCB signaling that is independent on dopamine D2 receptors or mGluR group 1 receptors, suggesting that these signaling pathways are required for facilitating a postsynaptic calcium increase to enable eCB production and release (Adermark and Lovinger, 2007; outlined in schematic drawing, Figure 5E). Quinpirole has previously been shown to robustly increase striatal release of the eCB anandamide in a dopamine D2 dependent manner (Giuffrida et al., 1999). Nicotine could thus facilitate eCB signaling by increasing dopamine D2 receptor signaling, thereby inhibiting cholinergic neurons, and reliving the muscarinic break on post synaptic calcium channels to promote eCB signaling (Wang et al., 2006; Adermark and Lovinger, 2007; Figure 5E). Furthermore, eCB may further suppress activity at striatal synapses by decreasing glutamate transporter activity, resulting in elevated extrasynaptic glutamate concentrations and an indirect suppression of presynaptic glutamate release (Brown et al., 2003; Adermark et al., 2022). While it remains to be determined, the data presented here suggest that nicotine increases eCB signaling, but that HFS is warranted to reach the threshold required for synaptic depression at excitatory terminals (Uchigashima et al., 2007; Adermark and Lovinger, 2009).

Importantly, while drugs of abuse produce synaptic plasticity mechanisms in the acute phase, repeated exposure has been shown to impair eCB signaling (Blanco et al., 2016; Wang et al., 2020). This holds true not only for nicotine (Baca et al., 2013; Xia et al., 2017; Adermark et al., 2019), but also for alcohol and cocaine (Adermark et al., 2011b; Wang et al., 2020). Especially, repeated nicotine exposure appears to impair dopamine D2R-dependent induction of LTD, rather than CB1R signaling *per se* (Baca et al., 2013; Adermark et al., 2019). In fact, HFS-LTD in male rats have been shown to be restored by quinpirole alone (Adermark et al., 2019), or by quinpirole in combination with the NMDA receptor antagonist APV (Xia et al., 2017). While the data presented here shows that pre-treatment with quinpirole enhance HFS-LTD in slices from vehicle-treated rats, it did not facilitate HFS-LTD in slices from rats receiving sub-chronic nicotine exposure. Interestingly, elevated levels of acetylcholine, which has been reported during nicotine abstinence (Rada et al., 2001), may transform dopamine D2 receptor signaling (Scarduzio et al., 2017). If this is a factor underlying the shifted response to quinpirole remains to be determined.

Repeated exposure to nicotine produced a robust behavioral sensitization, which is in agreement with previous studies (Benwell and Balfour, 1992; Ericson et al., 2010; Hamilton et al., 2014; Morud et al., 2016; Honeycutt et al., 2020). Behavioral sensitization to repeated psychostimulant administration has been proposed to reflect many of the neurochemical changes that are characteristic for drug addiction (Robinson and Berridge, 1993; Steketee and Kalivas, 2011), and is in this regard a well-established model to outline behavioral transformations elicited by drugs of abuse. Behavioral sensitization elicited by psychostimulants has been proposed to be driven by an interplay between dopaminergic and glutamatergic neuroadaptations (Robinson and Becker, 1982; Vezina, 1996; Kim and Vezina, 2002; Bamford et al., 2008; Yoon et al., 2008; Jing et al., 2018; Kim et al., 2022), and neuroplasticity in the dorsal striatum appears to be especially important for establishing these behavioral transformations (Durieux et al., 2012; Kim et al., 2022; Lotfi et al., 2022). The neurophysiological data presented here demonstrated a sensitized response to nicotine with respect to both dopaminergic-and glutamatergic signaling, thereby supporting a role for striatal neuroplasticity in behavioral sensitization. Part of these findings may be linked to increased number of nAChRs, which has been observed in the brains of experimental animals during nicotine treatment and in the brains of human smokers at autopsy (Benwell et al., 1988; Collins et al., 1994; Breese et al., 1997; Alasmari et al., 2021). However, the establishment of behavioral sensitization may also be linked to other forms of striatal neuroplasticity. In fact, inhibition of CB1R has been shown to enhance the motor stimulatory property of the dopamine D2 agonist quinpirole (Giuffrida et al., 1999). This finding suggests that the eCB system may act as an inhibitory feedback mechanism counteracting dopamine-induced facilitation of locomotion. This is especially interesting considering the finding that synaptic depression induced by acute nicotine exposure was potentiated in slices pre-treated with CB1R antagonist. In addition, the data presented here showed reduced HFS-LTD in response to dopamine D2 receptor activation in slices from rats receiving sub-chronic nicotine exposure. It is thus possible that impaired eCB signaling contributes the potentiation of behavioral and neurophysiological response to acute nicotine in rats receiving sub-chronic nicotine exposure.

While nicotine preferentially increases dopamine in the ventral as compared to dorsal striatum in animal studies (Imperato et al., 1986; Shim et al., 2001; Danielsson et al., 2021b), raclopride binding in dorsal striatum of humans have been shown to correlate with subjective measures of the rewarding properties of nicotine (Montgomery et al., 2007). Furthermore, when characterizing the effects of smoking on the mesolimbic dopamine system in humans, dopamine elevations in ventral striatum were consistent with studies performed in men, whereas dopamine responses in women were associated with subregions of dorsal striatum (Cosgrove et al., 2014). Nicotine may thus display sex-specific effects on striatal dopamine transmission. The data presented here show that nicotine elevates the microdialysate concentration of dopamine in the DLS, and that repeated exposure produces a sensitized response to the dopamine-elevating property of nicotine. The sensitized dopamine response may contribute to behavioral transformations and enhanced neuroplasticity in response to nicotine. While sensitized responses to nicotine-induced dopamine elevations have primarily been studied in the nucleus accumbens (Balfour et al., 1998; Vezina et al., 2007), our finding is in agreement with a previous study showing an increased dopamine elevation in dorsal striatum following local administration of nicotine (5 mM) in nicotine exposed rats (Shim et al., 2001). However, more research outlining dopaminergic neurotransmission in dorsal striatal subregions in females is warranted to outline putative sex-specific effects.

Abstinence to nicotine is associated with reduced dopamine levels (Rada et al., 2001; Rahman et al., 2004; Domino and Tsukada, 2009; Rademacher et al., 2016), and withdrawal-induced by the nAChR antagonist mecamylamine has been shown to produce a more pronounced decrease in dopamine levels in the nucleus accumbens of female compared to male rats (Carcoba et al., 2018). However, *in vivo* microdialysis performed in the DLS revealed no significant decrease in baseline dopamine levels at the time-point assessed here (5 days after the last nicotine injection). This finding may be associated with the fact that drug-induced changes in baseline dopamine levels are more pronounced in the nucleus accumbens compared to the dorsal striatum (Balfour et al., 1998; Danielsson et al., 2021a). In addition, findings may also be highly dependent on the time-point when dopamine levels were assessed (Hirth et al., 2016).

Repeated administration of nicotine resulted in a sustained suppression of evoked potentials in the DLS of female rats. This is partially in agreement with studies performed in male rats, although the neuroadaptations described here arise at an earlier time-point (Adermark et al., 2016). While increased eCB signaling in response to nicotine exposure might contribute to the sustained synaptic depression, decreased PS amplitude in nicotine abstinent rats was not associated with a change in PPR indicating that postsynaptic transformations may underly the sustained synaptic depression. While it remains to be determined what changes that underly the decrease in PS amplitude, impaired glutamate uptake may be one contributing factor. Both nicotine and eCB signaling impair glutamate uptake (Brown et al., 2003; Lim and Kim, 2003a,b; Alasmari et al., 2021), and elevated glutamate levels are reported following repeated nicotine exposure (Carcoba et al., 2018). Reduced glutamate clearance may thus contribute to both acute and sustained neuroplasticity elicited by nicotine (Adermark et al., 2022), possibly through remodeling of synaptic networks and post-translational modifications of glutamate receptors and their interacting proteins (Zheng et al., 2008; outlined in schematic drawing, Figure 5E).

Sex hormones may have the potential to modulate neurohormonal responses to nicotine and influence the psychoactive and reinforcing properties of the drug (Bertrand et al., 1991). Estradiol treatment decreased striatal dopamine D2 receptor binding, and variations in dopamine levels and binding are seen in different phases of the estrous cycle in rats (Bazzett and Becker, 1994; Al Sweidi et al., 2013). Still, there appears to be no estrous cycle dependent changes or sex differences when monitoring nicotine self-administration and reinstatement of nicotine-seeking (Feltenstein et al., 2012; Leyrer-Jackson et al., 2021). *In vivo* spontaneous activity of dopamine neurons, as well as *ex vivo* intrinsic and synaptic properties, have also been reported to be similar in male and female rats (Melis et al., 2013), even though there appears to be a sex difference with regards to tonic 2-AG signaling on to dopaminergic neurons (Melis et al., 2013).

There are several limitations with this study. Especially, electrophysiological recordings are conducted *ex vivo* and the reciprocal connection with other brain regions is thus lost. Field potential recordings also represent the collective activity of hundreds of neurons evoked by electrical stimulation, and differences in direct–indirect pathway neurons can thus not be assessed. Still, eCB-induced LTD is reported in glutamatergic synapses targeting both dopamine D1 and D2 expressing MSNs (Adermark and Lovinger, 2007). Another limitation is that plasticity in the DLS may be especially important during contingent administration and operant behaviors, but nicotine was administered in a non-volitional manner. While the data presented here demonstrates that nicotine produces acute and long-lasting effects on neurotransmission even without the involvement of instrumental learning, it is thus important to further evaluate nicotine-induced neuroplasticity following operant self-administration.

In conclusion, the data presented here demonstrates that nicotine facilitates eCB-mediated LTD and produces striatal synaptic depression by reducing the probability of transmitter release. These findings are similar to what has previously been reported in male rats suggesting that the acute effects mediated by nicotine on excitatory neurotransmission are independent on sex (Licheri et al., 2018; Adermark et al., 2019). However, a sustained depression of evoked PS amplitudes was observed at an earlier time-point in female rats, suggesting that they might be more susceptible to nicotine-induced neuroplasticity (Adermark et al., 2016). In addition, the acute effect of nicotine on PS amplitude was not occluded by dopamine D2 receptor activation, and D2 stimulation did also not facilitate HFS-LTD following sub-chronic nicotine exposure, which also disagrees with findings from male rats (Adermark et al., 2019). Considering the plastic interplay between dopamine and acetylcholine in eCB signaling and glutamate-induced synaptic depression (Calabresi et al., 1999; Wang et al., 2006; Adermark, 2011), these findings might be explained by the proposed sex specificity observed with regards to dopaminergic neurotransmission and dopaminergic regulation of reward-related behavior (Melis et al., 2013; Hynes et al., 2020, 2021). But more studies are required to fully establish sex-specific neuroplasticity associated with repeated nicotine exposure.

## Data availability statement

The raw data supporting the conclusions of this article will be made available by the authors, without undue reservation.

## Ethics statement

The animal study was reviewed and approved by Göteborgs djurförsöksetiska nämnd, Gothenburg Sweden.

## Author contributions

LA designed the study, conducted electrophysiological recordings, assembled figures, and drafted the manuscript. EL handled the animals, conducted behavioral experiments, and assisted during electrophysiological recordings. BS and ME assisted in data interpretation. All authors contributed to the article and approved the submitted version.

## Funding

This work was supported by the Swedish research council (vetenskapsrådet: Dnr: 2018‐02814, 2020-00559, 2020-01346, 2020-02105), and governmental support under the ALF agreement (ALFGBG-966287).

## Conflict of interest

The authors declare that the research was conducted in the absence of any commercial or financial relationships that could be construed as a potential conflict of interest.

## Publisher’s note

All claims expressed in this article are solely those of the authors and do not necessarily represent those of their affiliated organizations, or those of the publisher, the editors and the reviewers. Any product that may be evaluated in this article, or claim that may be made by its manufacturer, is not guaranteed or endorsed by the publisher.
